# Long-term effectiveness on glycemic control of insulin compared to combined oral antidiabetic drugs for initial intensive treatment in newly diagnosed type 2 diabetes: A duplicated target trial

**DOI:** 10.1097/MD.0000000000047235

**Published:** 2026-01-30

**Authors:** Tae Hyeon Kim, Yerin Hwang, Selin Woo, Kyeongmin Lee, Yejun Son, Seoyoung Park, Hyunjee Kim, Ju-Young Shin, YongHyun Cho, Dahye Shin, Dosang Cho, Kyung-Jae Lee, Sang Youl Rhee, Dong Keon Yon

**Affiliations:** a Center for Digital Health, Medical Science Research Institute, Kyung Hee University Medical Center, Kyung Hee University College of Medicine, Seoul, South Korea; b Department of Precision Medicine, Kyung Hee University College of Medicine, Seoul, South Korea; c Department of Medicine, Kyung Hee University College of Medicine, Seoul, South Korea; d Department of Regulatory Science, Kyung Hee University, Seoul, South Korea; e School of Pharmacy, Sungkyunkwan University, Suwon, South Korea; f RWE Division, Kakao Healthcare Corp., Seongnam, South Korea; g Department of Neurosurgery, College of Medicine, Ewha Womans University, Seoul, South Korea; h Department of Orthopaedic Surgery, Keimyung University Dongsan Hospital, Daegu, South Korea; i Department of Endocrinology and Metabolism, Kyung Hee University College of Medicine, Seoul, South Korea; j Department of Pediatrics, Kyung Hee University Medical Center, Kyung Hee University College of Medicine, Seoul, South Korea.

**Keywords:** insulin, metformin, oral antidiabetic drug, type 2 diabetes

## Abstract

Although both intensive insulin therapy (IIT) and combined oral antidiabetic drugs (COAD) can achieve initial intensive glycemic control, evidence comparing their long-term effectiveness following initial remission remains limited. Therefore, this study aims to compare long-term glycemic control and the risk of drug-free remission failure between IIT and COAD in patients who successfully achieved initial intensive glycemic control. This duplicated target trial utilized a cohort of 60,736 patients with type 2 diabetes from 3 hospitals in South Korea (2001–2024). Patients treated with insulin were assigned to the IIT group, and those receiving metformin combined with other oral antidiabetic drugs to the COAD group. Successful initial intensive treatment was defined as HbA1c <7.0% following treatment cessation. Failure of drug-free glycemic remission was defined as HbA1c ≥7.0% during follow-up. After 1:1 propensity score matching, Cox proportional hazard model was employed to evaluate the risk of remission failure between groups, estimating adjusted hazard ratios (aHRs) with 95% confidence intervals. Among 2092 patients achieving successful initial intensive treatment, each group assigned 355 following 1:1 propensity score matching (IIT group: mean age, 64.56 [standard deviation, 13.16] years; 49.0% female; COAD group: mean age, 64.08 [standard deviation, 12.90] years; 45.6% female). Baseline mean HbA1c and fasting plasma glucose were 6.26% and 115.4mg/dL in the IIT group, and 6.31% and 126.4mg/dL in the COAD group. During follow-up, HbA1c and body weight diverged significantly, with higher levels observed in the COAD group. The IIT group indicated a significant lower risk of drug-free glycemic remission failure compared to the COAD group (aHR, 0.47 [95% confidence interval, 0.31–0.73]). In patients with newly diagnosed type 2 diabetes achieving successful initial intensive treatment, IIT was associated with better long-term glycemic control compared with COAD, despite treatment discontinuation.

## 1. Introduction

Type 2 diabetes mellitus is characterized by insulin resistance, progressive β-cell dysfunction, and chronic hyperglycemia that increase the risk of microvascular and macrovascular complications.^[1]^ Persistent glucotoxicity compels pancreatic β-cells to sustain excessive insulin secretion, leading to intracellular oxidative stress and functional exhaustion. This β-cell fatigue accelerates the loss of insulin secretory capacity and further exacerbates hyperglycemia.^[2]^ Therefore, achieving rapid and effective glycemic normalization in the early stages of the disease can mitigate metabolic stress on β-cells, preventing dysfunction or apoptosis.^[3]^ Preserving β-cell function in this way may sustain endogenous insulin secretion for a longer duration, potentially delaying disease progression and enabling more stable long-term glycemic control.^[4]^

Evidence from landmark trials indicates that the long-term consequences of intensive control vary by population and treatment context. In newly diagnosed populations treated early to targets near 7%, posttrial follow-up in the United Kingdom Prospective Diabetes Study (UKPDS) showed sustained reductions in myocardial infarction and all-cause mortality, consistent with a legacy effect.^[5]^ By contrast, among higher-risk patients exposed to approximately 3.7 years of intensive control near normoglycemia, subsequent follow-up did not show durable reductions in major cardiovascular events or mortality.^[6]^ Intensive control consistently reduces microvascular outcomes,^[7]^ although the magnitude of benefit depends on clinical context. Collectively, prior evidence supports the biological plausibility of early intensive therapy, while indicating that the durability of benefit may depend on factors such as patient risk, treatment targets, and exposure duration.

In general, insulin therapy is reserved for individuals with marked hyperglycemia – such as those with HbA1c levels above 10% or blood glucose levels exceeding 300 mg/dL – whereas patients with subthreshold yet inadequately controlled diabetes are often treated with combination oral antidiabetic drug (COAD) regimens, typically including metformin as a backbone.^[8]^ In routine practice, the initial choice between insulin-based regimens and combination oral agents is often influenced by multiple clinical and practical factors, including glycemic severity, hypoglycemia risk, weight effects, tolerability, treatment burden, and cost, among others.^[8]^ Despite widespread use of both strategies as options for early intensive management, comparative evidence on their long-term effects on glycemic remission in newly diagnosed type 2 diabetes remains limited.

To address this gap, this study aims to emulate a target trial using real-world data to evaluate the long-term impact of initial intensive insulin therapy (IIT) versus combination oral antidiabetic drugs on glycemic remission in patients with newly diagnosed type 2 diabetes. By applying causal inference techniques within a target trial framework, this study seeks to generate robust and generalizable evidence to inform optimal early intervention strategies for type 2 diabetes management.

## 2. Methods

### 2.1. Data source

This study utilized clinical data from 3 major tertiary hospitals in South Korea: Kyung Hee University Medical Center (KHUMC), Keimyung University Dongsan Medical Center (KUDMC), and Ewha Womans University Medical Center (EWUMC). The data from each institution were standardized using the Observational Medical Outcomes Partnership Common Data Model (OMOP-CDM), which harmonizes electronic medical records and insurance claims data through a unified structure and terminology, enabling integrated analysis across diverse healthcare systems.^[9]^ The KHUMC OMOP-CDM database included electronic medical records data for 26,020 patients from 2019 to 2024; the KUDMC database included 12,372 patients from 2018 to 2024; and the EWUMC database contained 17,004 patients from 2001 to 2024.

### 2.2. Study design and population

**Figure 1.**
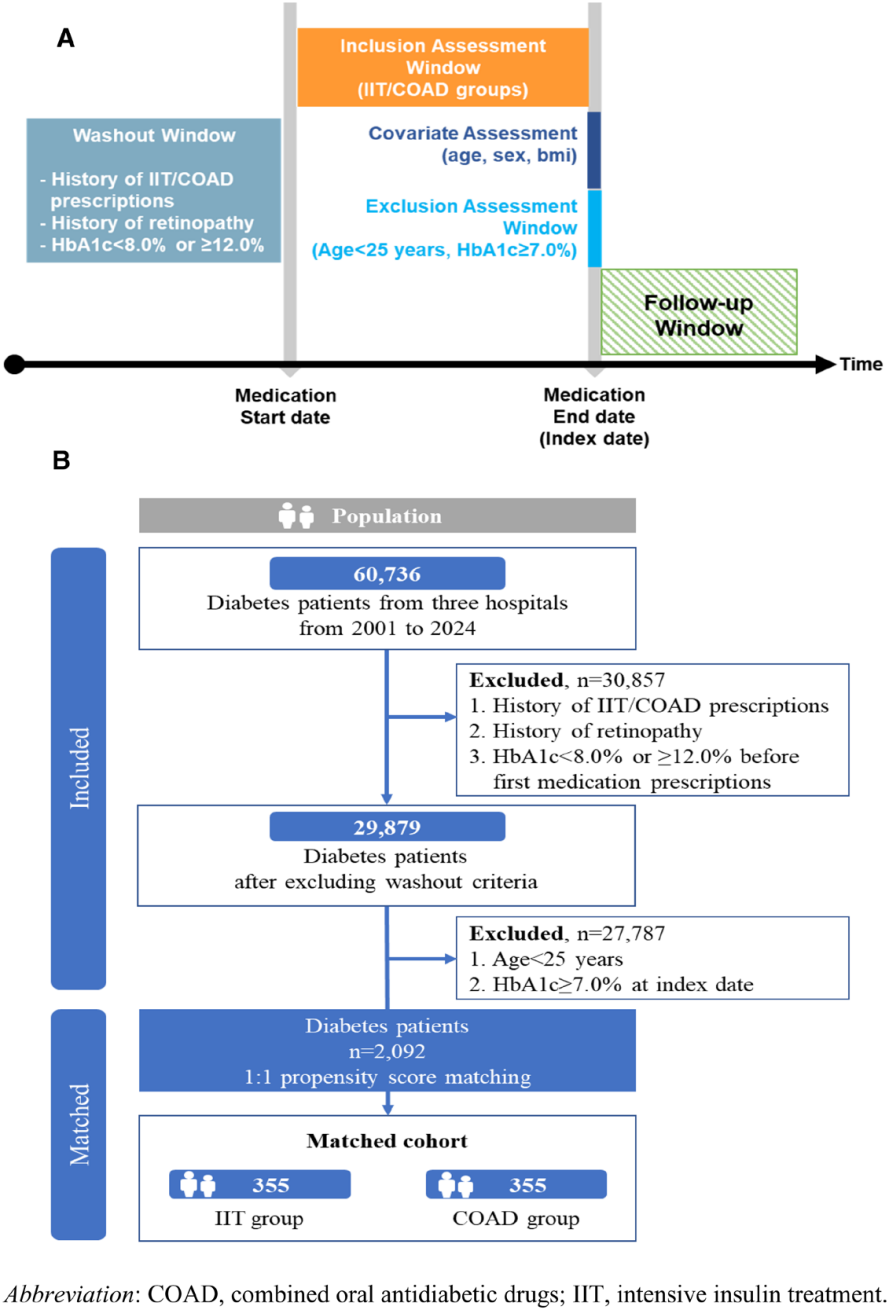
Design of study. (A) Study diagram and (B) flow chart of study population. COAD = combined oral antidiabetic drugs, IIT = intensive insulin therapy.

This study emulated a hypothetical target trial to evaluate the long-term effects on glycemic control based on the type of initial intensive therapy in patients with newly diagnosed type 2 diabetes. The cohort included patients from KHUMC, KUDMC, and EWUMC, identified between 2001 and 2024 (N = 60,736). In this study, patients with type 2 diabetes mellitus were identified from the OMOP-CDM database using standardized condition concept identifiers. Specifically, the standard SNOMED concept ID 201826, corresponding to “Type 2 diabetes mellitus,” was utilized. To ensure comprehensive inclusion, all descendant concepts of this identifier were also considered, encompassing related subtypes and complications within the SNOMED hierarchy. This approach aligns with established practices in OMOP-based research for accurately capturing type 2 diabetes cases. To identify patients with new-onset type 2 diabetes, those meeting the following exclusion criteria during the washout period were removed from the study (n = 30,857): history of antidiabetic medication prescription; history of diabetic retinopathy; and HbA1c <8.0% or ≥12.0% prior to the first medication prescription (Fig. 1). The protocol for this emulated target trial received approval from the institutional review boards of the KHUMC, KUDMC, and EWUMC (KHUH-2023-10-077, DSMC 2024-02-056, and SEUMC 2023-12-002-003) (Table S1, Supplemental Digital Content, https://links.lww.com/MD/R249). All procedures adhered to the ethical standards of the Declaration of Helsinki. As this was a retrospective study using anonymized data, participant consent was waived under the approved protocol.

### 2.3. Exposures and outcomes

Exposure refers to 1 of 2 types of initial intensive therapy: IIT, or COAD. Patients initiating antidiabetic medication for new-onset type 2 diabetes were considered to have received initial intensive therapy, which was considered to have ended if there was no prefill within 180 days of the last prescription.^[10]^ Antidiabetic medications were classified as IIT, representing insulin treatment, or COAD, which is a combination of metformin and other oral antidiabetic medication. Cases using both IIT and COAD simultaneously were excluded. A detailed list of antidiabetic medications included in our study provided in Table S2, Supplemental Digital Content, https://links.lww.com/MD/R249.

Initial intensive therapy was considered effective if HbA1c remained below 7.0% after its cessation.^[11]^ A total of 2092 patients were identified as having successfully completed initial intensive therapy, with HbA1c confirmed to be below 7.0% at the time of discontinuation. The study outcome is a failure to maintain HbA1c levels below 7.0% during the follow-up period.

### 2.4. Propensity score matching

To balance covariate distribution between the IIT and COAD groups, we employed 1:1 exposure-driven propensity score matching. The “greedy nearest-neighbor” algorithm was used for random selection without replacement within a caliper width of 0.001 standard deviations (SDs).^[12]^ Matching accuracy was assessed using standardized mean differences, with an SMD of <0.1 indicating a balanced distribution.^[13]^ The covariates matched included age, sex, body mass index, and baseline HbA1c.

### 2.5. Statistical analysis

Independent sample *t*-tests and Fisher exact or Pearson χ^2^ tests compared baseline and observation time point differences. Changes in body weight, fasting plasma glucose (FPG), and HbA1c were analyzed using repeated analysis of variance model or linear mixed models to evaluate group differences between IIT and COAD. Drug-free glycemic remission was assessed by Kaplan–Meier analysis, with significance determined by the log-rank test. Cox proportional hazards models evaluated the risk of remission failure between groups by estimating adjusted hazard ratio (aHR) along with 95% confidence interval (CI). All statistical analyses were conducted using SAS (version 9.4; SAS Institute Inc., Cary). A 2-sided *P*-value <.05 was considered statistically significant. Data visualizations were performed using R (version 4.4.2; R Foundation for Statistical Computing, Vienna, Austria).

### 2.6. Participant and public involvement

No study participants were involved in the research question or study design; the study was conducted independently of participant consultation. Results will be available to all participants or relevant communities upon request.

## 3. Results

In patients with new-onset type 2 diabetes who achieved an HbA1c level below 7.0% following initial intensive therapy (n = 2092), a 1:1 propensity score matching resulted in 355 individuals in both the IIT and COAD groups. The IIT group had a mean follow-up duration of 126.3 weeks (SD, 154.10), while the COAD group had a mean of 98.04 weeks (SD, 136.21). After initial intensive therapy, the IIT group showed a mean HbA1c of 6.26% (SD, 0.42) and a FPG 115.4 mg/dL (SD, 21.21), compared to the COAD group’s HbA1c of 6.31% (SD, 0.43) and a FPG 126.4 mg/dL (SD, 22.87). All covariates had an SMD <0.1, indicating successful matching and appropriate distribution between the 2 groups (Table 1).

**Table 1:** Baseline characteristics for 1:1 propensity score matched patients (IIT vs COAD).

	Unmatched patients			1:1 propensity score matched patients		
	IIT (n = 703)	COAD (n = 1389)	SMD	IIT (n = 355)	COAD (n = 355)	SMD
Follow-up period (wk), mean ± SD	127.5 ± 201.8	133.1 ± 232.2	0.026	126.3 ± 154.1	98.04 ± 136.21	0.051
Sex, n (%)						
Men	355 (50.5)	825 (59.4)	0.026	181 (51.0)	193 (54.4)	0.051
Women	348 (49.5)	564 (40.6)		174 (49.0)	162 (45.6)	
Age (yr), mean ± SD	64.08 ± 13.46	64.41 ± 12.58	0.026	64.56 ± 13.16	64.08 ± 12.90	0.096
BMI (kg/m^2^), mean ± SD	25.21 ± 4.01	25.47 ± 3.67	0.069	25.27 ± 4.05	25.51 ± 3.73	0.062
HbA1c (%), mean ± SD	6.18 ± 0.47	6.39 ± 0.40	0.488	6.26 ± 0.42	6.31 ± 0.43	0.098
FPG (mg/dL), mean ± SD	115.4 ± 20.84	121.4 ± 20.66	0.294	115.4 ± 21.21	126.4 ± 22.87	0.338

BMI = body mass index, COAD = combined oral antidiabetic drugs, FPG = fasting plasma glucose, HbA1c = hemoglobin A1c, IIT = intensive insulin treatment, SD = standrad deviation, SMD = standardized mean difference.

**Figure 2.**
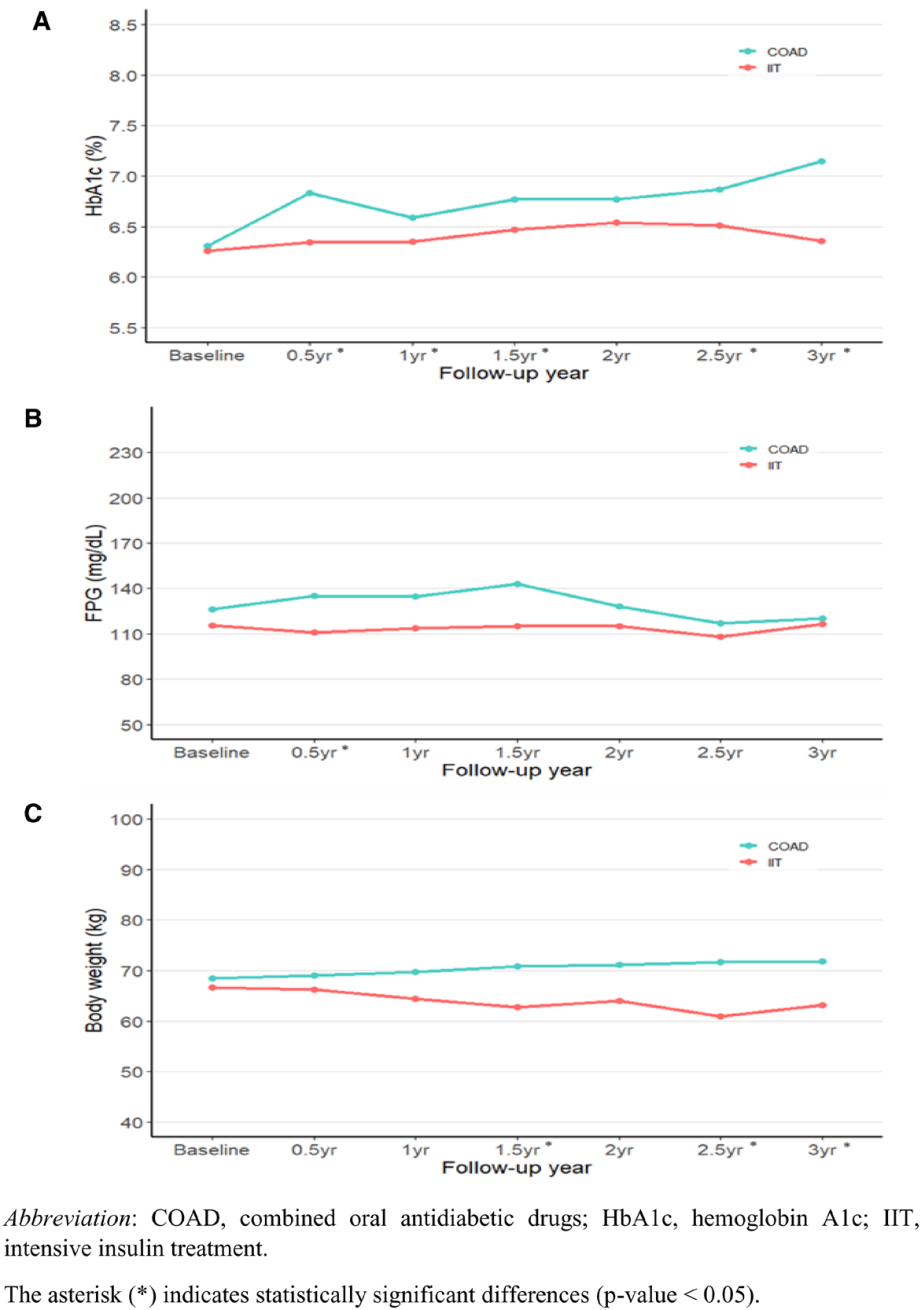
Mean HbA1c (A), fasting plasma glucose (B), and body weight (C) by treatment group over duration of study. The asterisk (*) indicates statistically significant differences (*P*-value <.05). COAD = combined oral antidiabetic drugs, HbA1c = hemoglobin A1c, IIT = intensive insulin therapy.

Figure 2 depicts changes in mean values of HbA1c, FPG, and body weight over a 3-year follow-up period following the cessation of initial intensive therapy. Although HbA1c and body weight were similar at baseline, the follow-up indicated an increasing divergence, with the COAD group showing higher values than the IIT group. For FPG, the differences between the 2 groups remained statistically insignificant throughout the follow-up, but the COAD group consistently showed higher mean values compared to the IIT group (Table S3, Supplemental Digital Content, https://links.lww.com/MD/R249).

**Figure 3.**
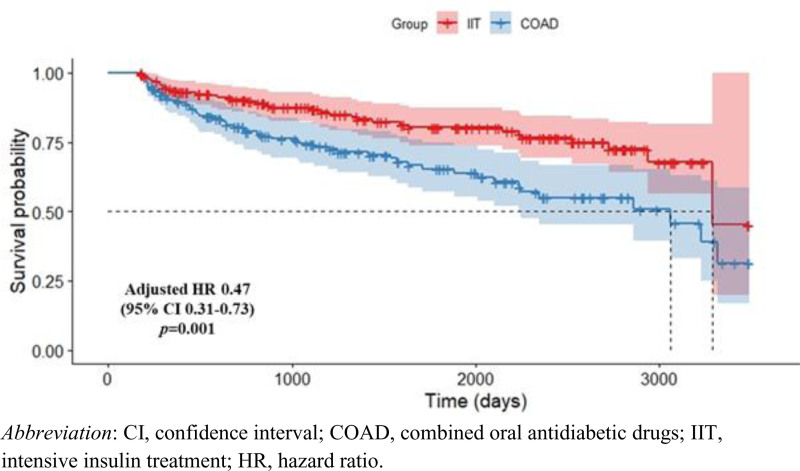
Survival of drug-free glycemic remission in IIT and COAD groups. CI = confidence interval, COAD = combined oral antidiabetic drugs, HR = hazard ratio, IIT = intensive insulin therapy.

During the follow-up period, HbA1c levels of 7.0% or higher were classified as failure of drug-free glycemic remission. The risk of failure was assessed and compared between the 2 initial intensive therapy approaches using a Cox proportional hazards model (Fig. 3). The IIT group indicated a significantly lower risk of failure compared to the COAD group (aHR, 0.41 [95% CI, 0.31–0.73]).

## 4. Discussion

### 4.1. Key findings

This emulated target trial investigated the long-term effects of glycemic control in patients with new-onset type 2 diabetes who successfully completed initial intensive treatment, comparing outcomes based on the type of treatment. The IIT group maintained significantly lower HbA1c and body weight than the COAD group after the cessation of initial intensive treatment. Notably, mean HbA1c in the IIT group remained below the 7.0% remission threshold even 3 years posttreatment. Defining failure of drug-free glycemic remission as HbA1c levels of 7.0% or higher during the follow-up period, the IIT group showed a significantly lower risk of failure compared to the COAD group. These results suggest that IIT was associated with more stable long-term glycemic control after the successful initial intensive treatment. Although the absolute HbA1c difference between the groups was modest, it persisted throughout the follow-up period, accompanied by consistently lower body weight in the IIT group. This sustained metabolic advantage suggests that early insulin-based intensive therapy may promote more durable glycemic stability beyond short-term remission.

### 4.2. Underlying plausible mechanism

Rapid glycemic control in newly diagnosed type 2 diabetes is clinically important, as early normalization of blood glucose reduces glucotoxicity, a key factor in long-term complications.^[14]^ This early intervention may facilitate recovery of β-cell function, which is vulnerable to chronic hyperglycemia’s toxic effects. By promptly alleviating glucotoxic stress through initial intensive treatment, residual β-cell function can be preserved or partially restored, promoting durable endogenous insulin secretion.^[15]^

Consequently, successful early glycemic control via IIT may lead to sustained glycemic remission and a lower risk of long-term complications.^[16]^ Evidence indicates that the duration and severity of prior hyperglycemia impact mortality more than current glycemic control levels, highlighting the need to minimize early metabolic damage.^[17]^ The concept of “metabolic memory” further supports the lasting effects of early glycemic normalization.^[18,19]^

IIT has shown particular effectiveness in achieving these outcomes, with studies reporting glycemic remission rates of 66.2% at 3 months and 46.3% at 12 months after initial intensive therapy and withdrawal of IIT.^[20]^ However, research on the long-term effects of initial intensive treatment using COAD is limited. One RCT found that COAD treatment with metformin/glimepiride had a higher risk of remission failure compared to IIT with glargine/glulisine, consistent with our findings.^[21]^

These observations align with previous evidence showing that sustained improvements in glycemic control confer long-term vascular benefits.^[22]^ In the UKPDS, each 1% reduction in HbA1c was associated with a 21% lower risk of any diabetes-related endpoint and a 37% reduction in microvascular complications.^[23]^ Consistent with these findings, population-based data from the Swedish National Diabetes Register identified elevated HbA1c as a strong predictor of myocardial infarction and stroke.^[24]^ Furthermore, meta-analyses of individual participant data from major intensive glucose control trials – including the Action to Control Cardiovascular Risk in Diabetes study, the Action in Diabetes and Vascular Disease: Preterax and Diamicron Modified Release Controlled Evaluation (ADVANCE) trial, UKPDS, and the Veterans Affairs Diabetes Trial – have shown that a mean reduction of approximately 0.9% in HbA1c corresponds to about a 9% lower risk of major adverse cardiovascular events.^[25]^ Collectively, these results reinforce that even modest but sustained HbA1c reductions can yield meaningful long-term vascular benefits.

### 4.3. Policy implications

Our findings indicate that initial intensive treatment with IIT in patients with newly diagnosed type 2 diabetes significantly increases the likelihood of sustained glycemic remission compared with COAD. Considering the established association between early glycemic control and long-term vascular outcomes,^[26,27]^ these results highlight the potential clinical value of timely intensive intervention for appropriate patients.

While evidence on the long-term effects of insulin versus oral antidiabetic agents remains inconsistent,^[28,29]^ our study suggests potential advantages of IIT. Although current guidelines typically recommend insulin for patients with markedly elevated HbA1c levels (≥10%), our findings suggest that earlier application of IIT could provide long-term metabolic benefits in selected patients. Policymakers and clinicians may consider exploring more personalized treatment strategies and reevaluating current thresholds based on patient characteristics and remission potential.

### 4.4. Limitations

This study has several limitations. First, despite emulating a target trial based on a well-structured protocol, we could not fully eliminate inherent biases common to observational studies, such as misclassification bias.^[30]^ Although this limitation is inherent to retrospective analyses, we minimized its impact by applying strict cohort definitions – identifying type 2 diabetes using SNOMED concept ID 201826 and its descendants, excluding patients with prior antidiabetic prescriptions, diabetic retinopathy, or baseline HbA1c outside 8.0 to 12.0% during the washout period – and by ensuring clear temporal relationships between treatment initiation, discontinuation (no refill within 180 days), and outcome assessment. Second, both IIT and COAD involve a variety of medications with differing dosages, which may impact treatment outcomes; however, our analysis did not differentiate by specific drug types or dosages. This heterogeneity could lead to underestimation of remission effects if certain COAD regimens (e.g., with weaker potency or poor adherence profiles) diluted the overall treatment effect compared with insulin-based therapy. While this may limit interpretability regarding individual agents, we focused on the broader treatment strategies (insulin *vs* COAD) to reflect real-world clinical decision-making, where medication regimens are often guided by institutional protocols and patient response. Third, according to Korean clinical guidelines, insulin is typically recommended for patients with HbA1c levels ≥10%, while COAD is preferred for those with less severe hyperglycemia.^[31]^ This raises the possibility of confounding by indication, whereby patients with more severe disease or poorer baseline control were more likely to receive IIT. Such bias would likely attenuate, rather than exaggerate, the observed benefit of IIT. To mitigate this, we employed propensity score matching to adjust for baseline differences including HbA1c. Fourth, the study population was restricted to Korean individuals, which may limit the generalizability of our findings to other ethnicities or healthcare systems. Differences in β-cell function, dietary composition, and treatment responses across populations may influence the durability of remission, underscoring the need for validation in more diverse cohorts. Nonetheless, the use of multicenter data from 3 major hospitals and standardized CDM-based methodology enhances the robustness and reproducibility of our findings across similar healthcare environments. Despite these limitations, this study’s strength lies in its emulation of a target trial design using a large, real-world cohort. Our approach provides evidence supporting the long-term benefits of IIT over COAD in patients with newly diagnosed type 2 diabetes who successfully achieved initial glycemic remission.

## 5. Conclusions

This emulated target trial suggests that initial intensive treatment was associated with achieving remission and maintaining long-term glycemic control in patients with newly diagnosed type 2 diabetes. Additionally, IIT was associated with more favorable long-term glycemic outcomes compared with COAD. Given that sustained hyperglycemia increases the risk of diabetes-related complications and mortality, early and effective glucose control remains an important clinical goal. Clinicians should acknowledge that insulin may offer greater efficacy for long-term outcomes in this patient population.

## Author contributions

**Conceptualization:** Tae Hyeon Kim, Yerin Hwang, Hyunjee Kim, Ju-Young Shin, Dahye Shin, Kyung-Jae Lee, Dong Keon Yon.

**Data curation:** Yerin Hwang, Ju-Young Shin.

**Formal analysis:** Yerin Hwang.

**Funding acquisition:** Dong Keon Yon.

**Methodology:** Yerin Hwang, Selin Woo, YongHyun Cho.

**Supervision:** Dahye Shin, Sang Youl Rhee, Dong Keon Yon.

**Validation:** Yejun Son, YongHyun Cho, Dosang Cho, Dong Keon Yon.

**Visualization:** Tae Hyeon Kim, Yerin Hwang.

**Writing – original draft:** Tae Hyeon Kim.

**Writing – review & editing:** Yerin Hwang, Selin Woo, Kyeongmin Lee, Yejun Son, Seoyoung Park, Hyunjee Kim, Ju-Young Shin, YongHyun Cho, Dahye Shin, Dosang Cho, Kyung-Jae Lee, Sang Youl Rhee, Dong Keon Yon.

## Supplementary Material


