# Supplementary material for: Long-term effectiveness on glycemic control of insulin compared to combined oral antidiabetic drugs for initial intensive treatment in newly diagnosed type 2 diabetes: A duplicated target trial
Source: Medicine (Baltimore). 2026 Jan 30;105(5):e47235. doi: 10.1097/MD.0000000000047235 (PMC12863771; doi:10.1097/MD.0000000000047235)
Supplement: Supplementary file 1 [file medi-105-e47235-s001.docx]

**Supplementary Table 1.** Emulated target trial framework evaluating long-term glycemic control effectiveness of IIT versus COAD in patients with successful initial intensive treatment of new-onset type 2 diabetes.

| Protocol component | Target trial specification | Target trial emulation |
| --- | --- | --- |
| Eligibility criteria | • Individuals newly diagnosed with type 2 diabetes, with HbA1c level below 8.0% or above 12.0%.  • Individuals who achieved an HbA1c reduction to below 7.0% following initial intensive treatment. | Same as for the target trial.  • We defined individuals with type 2 diabetes as those with SNOMED concept ID 201826, corresponding to “Type 2 diabetes mellitus.”  • To ensure inclusion of new-onset type 2 diabetes cases, participants were excluded if, during the pre-observation period, they had a history of antidiabetic medication use, diabetic retinopathy, or an HbA1c level below 8.0% or above 12.0% at the time of initial medication prescription.  • Initial intensive treatment was considered discontinued if no medication refill identified within 180 days. Patients achieving HbA1c level below 7.0% at discontinuation were classified as treatment successes and included in the study. |
| Treatment strategies | Patients with new-onset type 2 diabetes requiring glycemic control receive initial intensive treatment. | Same as for the target trial.  • Exposure to initial intensive treatment was defined by the presence of a prescription on the date of type 2 diabetes diagnosis. |
| Treatment assignment | Individuals are assigned to either the intensive insulin treatment (IIT) group or the combined oral antidiabetic drug (COAD) group. | Same as for the target trial.  • Patients initiating treatment with insulin (aspart, glulisine, and lispro) were assigned to the IIT group, while those prescribed metformin combined with other antidiabetic drug (acarbose, alogliptin, canagliflozin, dapagliflozin, empagliflozin, ertugliflozin, gemigliptin, gliclazide, glibenclamide, glimepiride, ipragliflozin, linagliptin, meglitinide, pioglitazone, rosiglitazone, saxagliptin, semaglutide, sitagliptin, vildagliptin, and voglibose) were assigned to the COAD group.  • Baseline covariates are assigned to adjust for confounding (age, sex, body mass index, and baseline HbA1c) |
| Outcomes | Failure of glycemic remission, which is defined as an HbA1c >7.0%. | Same as for the target trial. |
| Follow-up | • HbA1c, body weight, and fasting plasma glucose levels were tracked for up to three years from the date of initial intensive treatment cessation.  • Follow-up ends upon the occurrence of the outcome or at a maximum of three years. | Same as for the target trial  • The cessation date after successful initial intensive treatment was designated as the index date, with follow-up extending up to three years. |
| Causal contrast of interest | Intention-to-treat effect | Same as for the target trial |
| Statistical analysis | • Independent sample t-tests and Fisher’s exact or Pearson’s χ2 tests compared follow-up outcomes during the observation period to baseline values.  • Repeated measures ANOVA or linear mixed models compared the IIT and COAD groups over the follow-up period.  • The risk of drug-free glycemic remission failure between groups was assessed using Kaplan-Meier analysis and Cox proportional hazards model. | Same as for the target trial |

Abbreviations: ANOVA, analysis of variance model; COAD, combined oral antidiabetic drugs; IIT, intensive insulin treatment; SNOMED, Systematized Nomenclature of Medicine – Clinical Terms.

**Supplementary Table 2.** Medication composition of initial intensive treatment and combined oral antidiabetic drug regimens.

| **Drug** |
| --- |
| **Initial insulin treatment** |
| Aspart |
| Glulisine |
| Lispro |
| **Combined oral antidiabetic drugs** |
| Acarbose |
| Alogliptin |
| Canagliflozin |
| Dapagliflozin |
| Empagliflozin |
| Ertugliflozin |
| Gemigliptin |
| Gliclazide |
| Glibenclamide |
| Glimepiride |
| Ipragliflozin |
| Linagliptin |
| Meglitinide |
| Pioglitazone |
| Rosiglitazone |
| Saxagliptin |
| Semaglutide |
| Sitagliptin |
| Vildagliptin |
| Voglibose |

**Supplementary Table 3.** Mean HbA1c, FPG, and body weight by treatment group over duration of study

|  | **Baseline** | **~0.5 yrs** | **~1 yrs** | **~1.5 yrs** | **~2 yrs** | **~2.5 yrs** | **~3 yrs** | **p-value** |
| --- | --- | --- | --- | --- | --- | --- | --- | --- |
| **HbA1c, %** | | | | | | | | <0.001 |
| COAD | 6.31 | 6.83 | 6.59 | 6.77 | 6.77 | 6.87 | 7.15 |  |
| IIT | 6.26 | 6.34 | 6.35 | 6.47 | 6.54 | 6.51 | 6.36 |  |
| p-value | 0.119 | 0.005 | 0.010 | 0.010 | 0.087 | 0.011 | <0.001 |  |
| **FPG, mg/dl** | | | | | | | | 0.019 |
| COAD | 126.4 | 135.2 | 134.4 | 142.9 | 128.1 | 116.8 | 120.0 |  |
| IIT | 115.4 | 110.7 | 113.8 | 114.9 | 114.9 | 108.3 | 116.6 |  |
| p-value | 0.091 | 0.004 | 0.101 | 0.103 | 0.610 | 0.472 | 0.832 |  |
| **Body weight, kg** | | | | | | | | 0.341 |
| COAD | 68.41 | 68.98 | 69.77 | 70.85 | 71.06 | 71.61 | 71.83 |  |
| IIT | 66.71 | 66.25 | 64.41 | 62.69 | 64.00 | 60.86 | 63.10 |  |
| p-value | 0.460 | 0.538 | 0.115 | 0.008 | 0.061 | 0.003 | 0.018 |  |

Abbreviations: COAD, combined oral antidiabetic drugs; FPG, fasting plasma glucose; HbA1c, hemoglobin A1c; IIT, intensive insulin treatment.
